# Activity of beavers as an ecological factor that affects the benthos of small rivers - a case study in the Żylica River (Poland)

**DOI:** 10.2478/s11756-018-0073-y

**Published:** 2018-06-11

**Authors:** Małgorzata Strzelec, Katarzyna Białek, Aneta Spyra

**Affiliations:** Department of Hydrobiology, Faculty of Biology and Environmental Protection, University of Silesia, Bankowa 9, 40-007 Katowice, Poland

**Keywords:** Beaver dam, Invertebrate fauna, Mountain rivers

## Abstract

The Eurasian beaver (*Castor fiber*) has a greater impact on local ecosystems than other herbivores, and the affected area exceeds the range of its presence. Its activity may change or create new habitats by modifying the availability of the biotic and abiotic resources available not only to beavers, but also to other groups of animals. Our aim was to study how beaver activity affects the benthos composition in a small river and in a beaver pond. The way in which beavers function in the case of small rivers has received little attention. The study showed a lower density of benthos above the dam (beaver pond - 1467 ind./m^2^) compared to the river (3147 ind./m^2^). Below the dam, the diversity of Trichoptera and Coleoptera was greater, while Diptera were more abundant in the beaver pond. Betidae were a constant component in the benthos assemblages and were most abundant in the beaver pond. Collectors-gatherers and predators were the most numerous in the beaver pond. Decreasing percentages of collectors-gatherers were observed with an increased abundance of collectors-filterers and shredders in the river. All of the ratio values except the P/T FFG (Predators to total of all other groups) were lower in the beaver pond compared to the river. The % EPT (Ephemeroptera Plecoptera and Trichoptera) was also greater in the beaver pond. Small differences in the physicochemical properties of the water and organic matter content in the bottom sediments were found both above and below the beaver dam.

## Introduction

The most characteristic indicator of the presence and activity of beavers are their dams, which may be constructed as a single threshold or as a cascade. They are primarily built on small streams or between ponds in places that have a relatively constant level of water where beavers have year-round access to the water resources. The main factor that motivates beavers in their construction activity is safety (Żurowski 1992), but in specific cases, the sound of the water stimulates them to build dams (Hartman and Rice 1963; Johnston 2017). Beavers are mediators of landscape recovery, that create an increase in the ecological stability of the land (Gurnell 1998; Miranda 2017) and their activity alters a habitat and makes it unique (McDowell and Naiman 1986; Kusztal et al. 2017). No mammal species is characterised by such a wide range of construction-constructive behaviours (Żurowski 1992; Nummi et al. 2011).

The Eurasian beaver (*Castor fiber* Linnaeus, 1758) is a legally protected species in most European countries due to the legislation that defines its protection: the Convention for the Conservation of European Wildlife and habitats and the Council Directive 92/43/EEC on the Conservation of natural habitats and of wild fauna and flora (Annexes II and V). In Poland, it is a partially protected species (Bereszyński and Homan 2007; Dz. U 2011) and currently, according to the Regulation of Ministry of Environment from 16 December 2016 on the protection of species of animals, it is possible to hunt beavers from October 1 to March 15 (Dz. U 2016). In Poland, their presence has been reported in about 54% of the rivers, 29% of the lakes and 17% of the peat excavation sites and mid-forest swamps, except in the higher mountains (Janiszewski et al. 2009; Święcicka et al. 2014). The project of the beaver’s reintroduction intensified its spread in Poland (Żurowski 1979; Graczyk 1990; Czech and Lisle 2003; Janiszewski et al. 2009; Wajdzik et al. 2013). In the southern part of Poland, beaver populations are relatively less numerous compared to other parts of the country, e.g., north-eastern Poland (GDOŚ 2015).

The Eurasian beaver has a greater impact on the local ecosystems that it occupies, than other herbivores (Johnston and Naiman 1987; Stringer and Gaywood 2016; Larsen et al. 2017), and the area that is affected, exceeds the range of their distribution and existence (Czech 2007). They are known as potentially important ecosystem engineers (Wright and Jones 2006; Arndt and Domdei 2011; Peterrson and Schulte 2016). Their activity may change or create new habitats that are not only available to beavers (Rosell et al. 2005; Baker et al. 2012; Pliūraitė and Kesminas 2012), but also to other animal groups such as amphibians (Cunningham et al. 2006; Vehkaoja and Nummi 2015), fishes (Schlosser and Kallemeyn 2000; Janiszewski et al. 2014), small mammals (Nummi et al. 2011; Suzuki and McComb 2004), and also many species of birds. This is possible due to their extensive ecotone zone, which includes the presence of dead trees, islets and branches (France 1997).

The activity of beavers contributes to an increase in the surface and groundwater retention, which has an influence on stream morphometry (Naiman et al. 1986; Smith et al. 1989; Westbrook et al. 2006; Grygoruk and Nowak 2014). The proper functioning of a dam also reduces the severity of flooding events. Moreover, the reduced water-flow and the creation of lentic environments (beaver ponds) (Smith et al. 1991) slow down the erosion processes (Butler and Malanson 2005; Rosell et al. 2005). The scale of these type of changes depends on the size of the river (Kobojek 2013). Dams effectively reduce the water flow, whereas the created ponds play a role in the purification of the dammed water and in the deposition of organic matter and sediments (Naiman et al. 1986; Butler and Malanson 1995, 2005), which includes not only the river material, but also the mineral material from the process of the excavation of a burrow (Butler and Malanson 1995; Pliūraitė and Kesminas 2012). The activity of beavers has an impact on the physico-chemical properties of the water in a river and beaver ponds (Arndt and Domdei 2011; Szpikowska and Szpikowski 2012; Fracz and Chow-Fraser 2013; Čanády et al. 2016). The neutralisation of the pH retention of nutrients and heavy metals and a reduction of the oxygen content in the water (Lazar et al. 2015) are characteristic for beaver ponds compared to the adjacent river. The cut down trees is one of the consequences of beaver activity and this causes an increased exposure of the water to the sunlight (Naiman et al. 1986). In the case of shallow and extensive ponds, this lead to an increase in their water temperatures, which can consequently increase the temperature of the river water below the pond (Margolis et al. 2001). In small rivers, however, the small surface area of beaver ponds does not change the water temperature above and below the dam, because the water seeps over the entire surface of the dam and thus does not disturb the thermal regime of the watercourse (McRae and Edwards 1994). The engineering activities of beavers influence the physicochemical properties of the water and bottom sediments (Skinner et al. 1984; Margolis et al. 2001; Rosell et al. 2005), although the size and the nature of changes in the physicochemical properties is determined by the catchment type (Rosell et al. 2005).

In the beavers’ habitat, their food preference can have a significant impact on the course of the succession, species composition and structure of the plant communities (Rosell et al. 2005; Czech 2007). More than 86 species of trees and 149 herbaceous plants are eaten by beavers (Jenkins 1979; Dvořák 2013). Although in some habitats, water plants comprise 60–80% of their diet, they may be an important part of the diet of beavers due to the differences in their abundance (Stringer and Gaywood 2016). The presence of aquatic and rush vegetation and their remains has an impact on the occurrence of benthic fauna. Our aim was to study how beaver activity affects the benthos fauna composition in a beaver pond and in a small river. Little attention has been paid to the way in which beavers function in small rivers. The following hypotheses were constructed: 1. The activity of beavers is an important ecological factor that influences the benthos of a small river; 2. The community composition of benthos and functional feeding groups will differ between the river course and in the beaver pond as a consequence of differences in their environmental factors such as a slow current and lentic conditions.

## Material and Methods

### Study area and sampling procedure

**Fig. 1:**
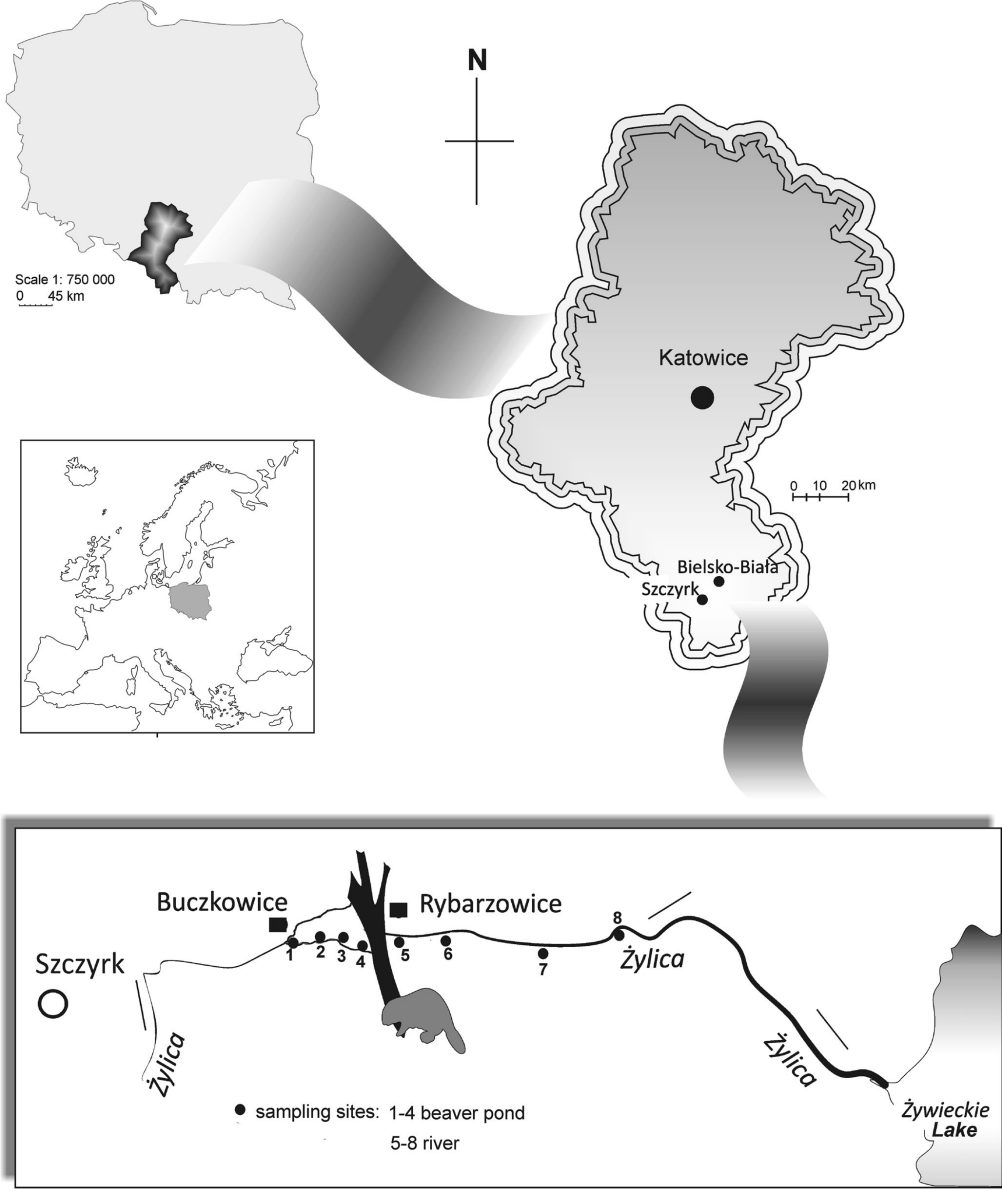
Location of the study sites and the beaver dam in the Żylica River

The study was carried out in the small mountain river – the Żylica River (a total length of 21.8 km) (Fig. 1), which is a left-bank tributary of the Soła River. Its source is located at 1050 m a.s.l., whereas the mouth of Żywieckie Lake (one of the dam reservoirs in the Soła cascade) lies at 342 m a.s.l. The river flows through urban areas, forests and meadows. In most parts, especially in the urban areas, the Żylica River is regulated through the technical regulation of the river bed and shores (Janus et al. 2009). The hydromodifications and technical elements include the water threshold, which makes passage along or across rivers easier, as well as the construction of weirs and technical structures (Janus et al. 2009).

The sampling sites were selected in the middle course of the river at a distance from urban buildings and communication routes, in places where the river banks have not been developed for tourists nor used economically. The sampling sites were selected according to their location with respect to the beaver dam, four sampling sites were selected above the dam within the area of the beaver pond (pond cover 4969.2 m^2^) (from N 49°43.625′, E 19°05.846′ to N 49°43.622′, E 19°05.971′) and four were selected below a beaver dam in a natural part of the river (from N 49°43.615′, E 19°05.984′ to N 49°43.623′, E 19°06.181′). The sampling sites were selected at a distance of between 40 and 110 m in the beaver pond, and 66–160 m in the river. The shoreline of the river is covered with deciduous trees, which are predominantly *Alnus glutinosa*, *Fraxinus excelsior*, *Salix alba*, and *Salix purpurea* and shrubs.

The width of the river at the beginning of the pond is from 16.2 m (site 1) to 24.26 m. (site 4). The bottom is covered with stones and different thicknesses of mud (increasing along the distance to the dam) and leaf deposits from – *A. glutinosa*, *F. excelsior*, *S. alba* and *S. purpurea*. During the study period, traces of the presence of beavers, following their engineering activities in the form of stumps of deciduous trees, as well as bitten branches that were submerged in the water, was observed in area of the beaver pond.

The width of the river below the beaver dam ranged from 8.79 m (site 8) to 13.53 m (site 6). The bottom at the sampling sites located below the dam consisted of stones, mud, branches and leaves from the riparian trees as well as macrophytes. The depth of the water at the sampling sites was different and ranged from 0.18 m (site 5 and 6) to 0.53 m (site 8). The banks of the river were overgrown with neophytes e.g., *Reynnoutria* sp., which formed dense phytocenoses just off the banks (sites 3 and 8). At the other sampling sites *Lysimachia nummularis* and *Phragmites australis* and *Phalaris arundinacea* (site 1) occurred. The river banks were bushy, which resulted in a reduced heating of water and the bottom was covered with boulders and rock faults. The selection of the sampling sites, taking into account the nature of the river, permitted representative benthic invertebrate material to be collected.

The samples were taken in the Żylica River during the summer and autumn of 2015 at each of the eight sampling sites that were selected along its course. The periods of sampling were determined according to the guidelines for monitoring animals (Makomaska-Juchiewicz and Bonk 2005). Benthic invertebrates were sampled from a 0.25 m^2^ bottom area from all substrates, using a quadrat frame and a hydrobiological net (multihabitat approach). The frame was dipped into the substratum four times (four subsamples) on each of the study sites. The samples were transported to the laboratory and sieved on 0.23 mm mesh size sieves. Live invertebrates were selected from the samples using a Stereoscopic Microscope, preserved in 75% ethanol, identified and counted. The number of individuals was estimated as the invertebrate density (ind./m^2^). In most cases, the invertebrates were identified to the family level (except molluscs) according to Rozkošný (1980) and Piechocki and Wawrzyniak-Wydrowiska (2016).

Macrophytes were identified in both the beaver pond and the river sampling sites and identified to the species level according to Szafer et al. (1986). The width of the river and the water velocity were also measured at each of the sampling sites in the field. Along with the invertebrate sampling, water samples were also taken in order to measure the content of nitrates, nitrites, ammonia, phosphates, calcium and chlorides and the total hardness (Hanna Instruments meters, Merck titrimetric and colorimetric methods) as well as the total dissolved solids (TDS), pH and conductivity and temperature (field measurement, Hanna Instruments HI 9811–5 electrode). The content of dissolved oxygen was measured in the field with oxygen meter. The methods for the analysis of water chemistry were adopted according to Hermanowicz et al. (1999).

At each site, samples of the sediment were also taken in order to determine the organic matter content. The loss on ignition (LOI) method was used according to PN-88/B-04481 (Myślińska 2001). The content of organic matter in the bottom sediments was described according to the classification of Verdonschot (2001): >10.0%- very high, 4.1–10.0% -high, 1.0–4.0%- medium and < 0.1% low organic matter content.

### Data analysis

The structure of the benthos communities were analysed using the following indices:

1. Constancy (%) according to Górny and Grüm (1981) - the values were divided into four classes: euconstants 75.1–100%, constants 50.1–75.0%, rare taxa 25.1–50.0, accidental taxa ≤25.0
2. The Shannon-Wiener index (H′) (Krebs, 1989; McCune, Grace, & Urban, 2002)

A functional feeding groups (FFG) classification was developed as a tool to facilitate the incorporation of invertebrates in the aquatic lotic environments. Benthic insects were divided into the FFG according to Merritt and Cummins (1978, 1996), and Cummins et al. (2005). Five FFG of insects were used: predators, collectors-gatherers (gathering collectors), collectors-filterers (filtering collectors), shredders and scrapers. We selected four functional feeding type indices (Table 1). The ratios for the Predators to the total of all of the other groups (P/T FFG), Filtering Collectors to Gathering Collectors (FC/GC), Shredders to total Collectors (SH/TC) as well as Scrapers + Filtering Collectors to Shredders + Gathering Collectors (SC FC/ SH GC) were determined according to Merritt et al. (1996) as indicators of the attributes of the stream ecosystem. The ratios are responsive to changes in the food base and are a useful ecological tool, that indicates various ecosystem parameters (Redin and Sjöberg 2013).

**Table 1:** The values of the Functional Feeding Group (FFG) ratios and the EPT indices at the sampling sites located above (beaver pond) and below the beaver dam (river)

	Beaver pond				River				T-test	*p*
	1	2	3	4	5	6	7	8		
FC/GC*	0.02	0	0.003	0.02	0.08	0.81	0.74	0.73	3.387	0.001
SH/TC	0.01	0	0.02	0.02	0.006	0.01	0.03	0.03	0.813	0.440
SCR F/ SHR GC*	0.03	0	0.003	0.03	0.08	0.79	0.72	0.76	3.363	0.001
P/T FFG	0.26	0.29	0.16	0.15	0.02	0.28	0.09	0.28	0.694	0.510
% EPT*	45.69	51.4	39.4	35.5	6.56	16.56	15.15	8.24	7.311	0.0003
EPT taxa*	0.29	0.12	0.15	0.17	0.44	0.23	0.38	0.37	2.973	0.020

*- significant differences using the t-test, FC/GC - Filtering Collectors to Gathering Collectors, SH/TC - Shredders to total Collectors, SC FC/SHR GC - Scrapers + Filtering Collectors to Shredders + Gathering Collectors, P/T FFG - Predators to total of all other groups, EPT – Ephemeroptera + Plecoptera + Trichoptera

We calculated the EPT as the percentage of individuals in the insect orders Ephemeroptera, Plecoptera, and Trichoptera (E + P + T/Total benthos × 100% = EPT %) and the EPT taxa as the total number of the taxa Ephemeroptera, Plecoptera, and Trichoptera (number of E + P + T families/total number of benthos families).

Cluster analysis of the benthic invertebrates on the sampling sites was assessed using a hierarchical analysis using the Unweighted Pair-Group Average Method (UPGMA) and Euclidean distance. A dendrogram of faunistic similarities was obtained using the MVSP program version 3.13p.

The significance of the differences in the density of the benthos fauna, the number of taxa, the Shannon index and the FFG ratios between the sampling sites was evaluated using the Student’s T- test (using the Kolmogorov-Smirnov test for normality). EPT taxa were log transformed (log10 n + 1). A significance level of *p* < 0.05 was specified for all of the statistical analyses (STATISTICA 12.0).

## Results

### Environmental characteristics

Small differences in the physicochemical properties of the water and the organic matter content in the sediments were found at the sampling sites selected for this study (Table 2). At the sampling sites located above and below the beaver dam, the organic matter content was similar (high or very high). Aquatic vegetation was rather scarce with several species occurring at a few of the sites e.g., *Phragmites australis* and *Phalaris arundinacea* (site 1), *Lysimachia nummularia* (site 2), *Reynoutria japonica* (site 3, 7 and 8) and *Humulus lupulus* (site 8).

**Table 2:** Water chemistry, organic matter content and velocity above and below the beaver dam

	Beaver pond	River
OM^a^ %	4.43–21.9	3.92–31.57
Temperature °C	5.5–15.5	5.65–15.1
pH	7.1–8.0	7.5–8.5
Conductivity μS cm^−1^	140–190	130–195
TDS mg dm^−3^	60–90	60–90
Dissolved oxygen mg dm^−3^	5.65–12.1	7.5–12.8
Nitrate mg dm^−3^	6.6–14.2	10.03–26.58
Nitrite mg dm^−3^	0.0–0.13	0.0–0.01
Ammonia mg dm^−3^	0.23–0.28	0.18–0.72
Phosphate mg dm^−3^	0.0–0.07	0.0–0.18
Total hardness mg CaCO_3_ dm^−3^	58.9–105	42.8–105
Calcium mg dm^−3^	21–26	18–29
Chlorides mg dm^−3^	10–14	10–18
Velocity m s^−1^	0.0	0.08–0.15

^a^ Organic matter content in the bottom sediments

The water chemistry did not differ significantly between the sampling sites in the beaver pond and in the river. While the greatest differences were observed in relation to the nitrate and ammonia content in the water, they were not statistically significant.

### Benthos composition and spatial distribution

A total of 56 taxa were found in the benthos - 43 in the beaver pond and 50 in the river (Table 3). The mean density of the benthos was smaller in the beaver pond (1467 ind./m^2^) compared to the river (3147 ind./m^2^) (*t* = 3.611, *p* = 0.01). In the downstream part of the river, the diversity of Trichoptera (10 taxa) and Coleoptera (6 taxa) was greater, as opposed to higher diversity of Diptera in the beaver pond.

**Table 3:** Composition of the benthos assemblages in the beaver pond and the downstream part of the Żylica River

Taxa		Beaver pond		River	
		%^a^	C^b^	%^a^	C^b^
Gastropoda	Lymneaeidae (juv.^c^)	0.58	50	0.08	37.5
	Physidae (*Physa acuta* Draparnaud, 1805)	0.07	12.5	0.06	25
	Planorbidae (*Gyraulus albus* O. F. Müller, 1774)	0.07	12.5	0.68	100
Bivalvia	Sphaeriidae (*Pisidium* sp.)	1.91	75	3.22	87.5
Oligochaeta	Oligochaeta	2,21	50	1.43	87.5
Hirudinea	Erpobdellidae	0.34	62.5	2.58	87.5
	Glossiphoniidae	–	–	0.36	62.5
Crustacea	Gammaridae	10.3	100	26.5	100
	Asellidae	–	–	0.68	37.5
Plecoptera	Leuctridae	0.03	12.5	0.24	50
	Perlidae	–	–	0.01	12.5
	Nemouridae	0.2	25	0.49	37.5
	Coenagrionidae	0.61	75	0.05	12.5
Odonata	Platycnemididae	0.03	12.5	0.19	50
	Calopterygidae	0.07	12.5	0.19	37.5
	Aeshnidae	–	–	0.08	25
Ephemeroptera	Baetidae	41.45	100	4.27	87.5
	Leptophlebiidae	0.14	25	0.79	75
	Caenidae	0.51	25	0.14	50
	Heptageniidae	0.03	12,5	0.33	25
	Ephemeridae	–	–	0.01	12.5
Megaloptera	Sialidae	–	–	0.03	25
Coleoptera	Dytiscidae	3.58	100	1.08	62.5
	Elmidae	3	87.5	29.2	100
	Helodidae	0.07	25	0.03	25
	Haliplidae	0.54	50	0.03	25
	Gyrinidae	–	–	0.03	25
	Chrysomelidae	–	–	0.01	12.5
	Hydrophilidae	0.1	25	–	–
Trichoptera	Polycentropodidae	0.24	50	0.65	87.5
	Goeridae	0.03	12.5	0.03	25
	Leptoceridae	0.51	62.5	1.73	62.5
	Limnephilidae	–	–	0.3	62.5
	Hydropsychidae	0.07	12.5	1.1	25
	Rhyacophilidae	–	–	0.08	25
	Phryganeidae	–	–	0.03	12.5
	Sericostomaidae	–	–	0.24	37.5
	Psychomyidae	–	–	0.06	37.5
	Beraeidae	–	–	0.06	12.5
Heteroptera	Corixidae	9.06	100	2.56	75
	Veliidae	0.48	62.5	1.62	62.5
	Mesovelidae	0.07	12.5	–	–
	Notonectidae	0.14	50	0.05	37.5
	Gerridae	0.17	25	0.01	12.5
	Nepidae	0.03	12.5	0.01	12.5
Diptera	Chironomidae	21.8	100	11,09	100
	Dixidae	0.20	50	–	–
	Ceratopogonidae	0.37	37.5	0.32	50
	Tabanidae	0.03	12.5	0.06	25
	Culicidae	0.34	25	–	–
	Limoniidae	0.14	25	0.11	37.5
	Tipulidae	0.07	12.5	0.06	25
	Psychodidae	0.24	37.5	0.08	25
	Simuliidae	0.03	12.5	0.05	12.5
	Syrphidae	0.03	12.5	–	–
	Stratiomyidae	0.03	12.5	–	–
Mean density (ind./m^2^) / SD					
Number of taxa		1467/195.28		3147/450.42	
Shannon-Wiener index		43		50	
at sampling sites (min-max)		2.1–3.1		1.9–3.4	

^a^relative abundance (percentage share), ^b^ Constancy index (%), ^c^- juvenile specimens

Betidae were a constant component in the benthos assemblages among Ephemeroptera and they were most abundant in the beaver pond. Gammaridae and Chironomidae were characterised by the highest value of the Constancy index (C = 100%, euconstants) both above and below the beaver dam. Six taxa were only found in the beaver pond e.g., Hydrophilidae, Mesovelidae, Dixidae, Culicidae and Syrphidae, whereas 14 taxa were found in the river (Table 3). Taking into account the values of the Constancy index, in both the beaver pond and in the river, they constituted a rare or accessory components in the benthic fauna and their percentage did not exceed 1%. However, below the beaver dam (at the river sampling sites), species that are sensitive to pollution and characteristic for the lotic ecosystems such as Sericostomatidae, Bereidae, Ryacophilidae and Limnephilidae were found. Gammaridae, Chironomidae and also Betidae constituted more that 10% of the benthos fauna in the beaver pond. In the river more that 10% of the collection comprised Gammaridae, Chironomidae and Elmidae (Table 3). Although the richness and the number of taxa were different above and below the dam, the values of the Shannon-Wiener diversity index did not differ significantly.

**Fig. 2:**
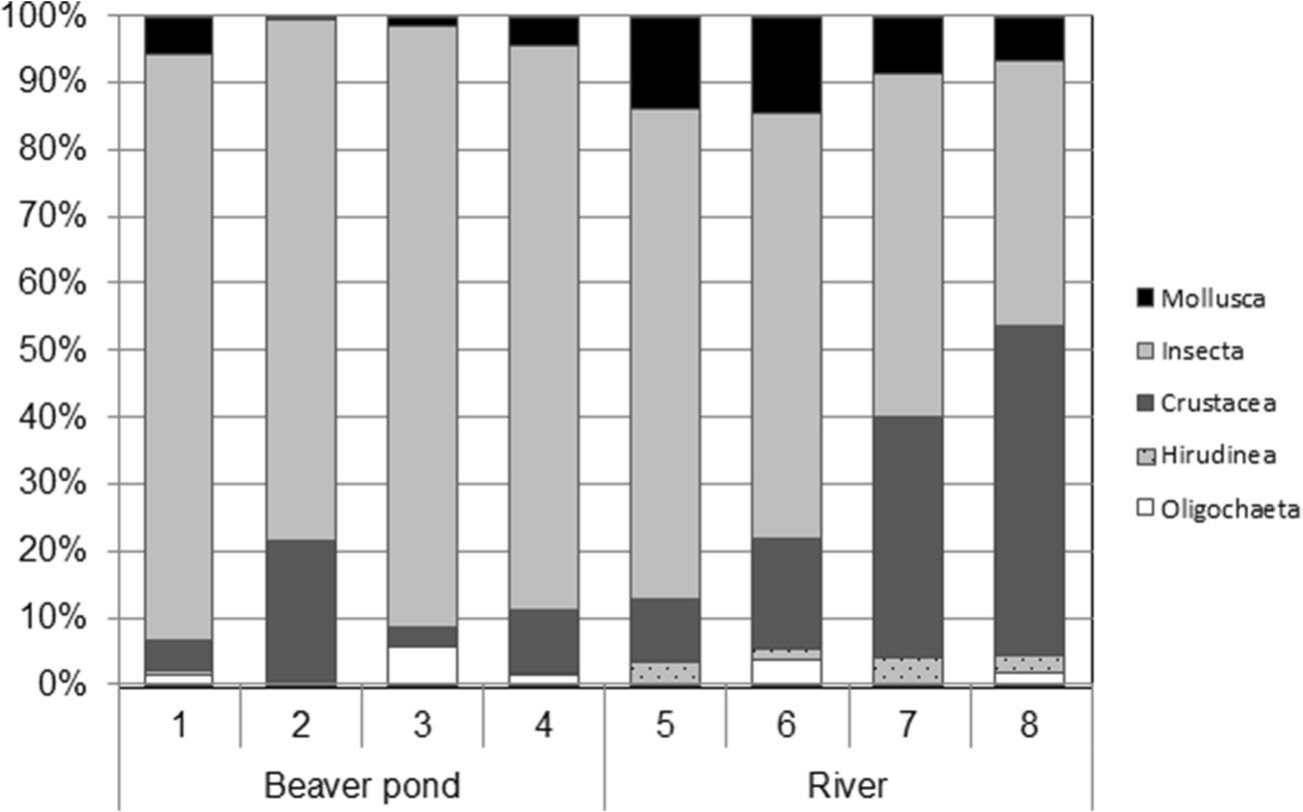
Percentage share of the main benthos taxa groups in the beaver pond (sites 1–4) and in the river (sites 5–8)

The composition of the benthos collected at the river sampling sites and in the beaver pond were different (Fig. 2). At the sites that were located in different parts of the beaver pond, the most numerous taxonomic group in the benthos fauna were insect larvae, while on site 2, crustaceans comprised 20% of the benthic fauna. In the downstream part of the river, a progressive increase in the proportion of crustaceans was observed. Molluscs and leeches were more frequent in the river compared to the pond (Fig. 2, Table 3).

The % EPT was visibly greater in the beaver pond, which was the consequence of the numerous occurrence of Betidae larvae. The EPT taxa was higher in the river due to the greater number of Ephemeroptera, Plecoptera and Trichoptera taxa (Table 1).

**Fig. 3:**
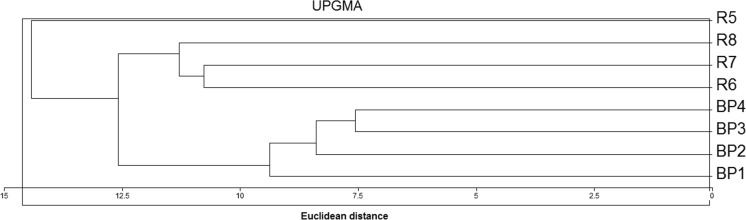
Dendrogram of the faunistic similarities of the benthos fauna in the beaver pond (sites BP1–4) and in the river sites R5–6)

Cluster analysis grouped the sampling sites from the beaver pond into one group and three typically riverine sampling sites into another. As is visible on Fig. 3, the site located just below the beaver dam (site 5) is clearly distinct.

### Functional feeding groups

**Fig. 4:**
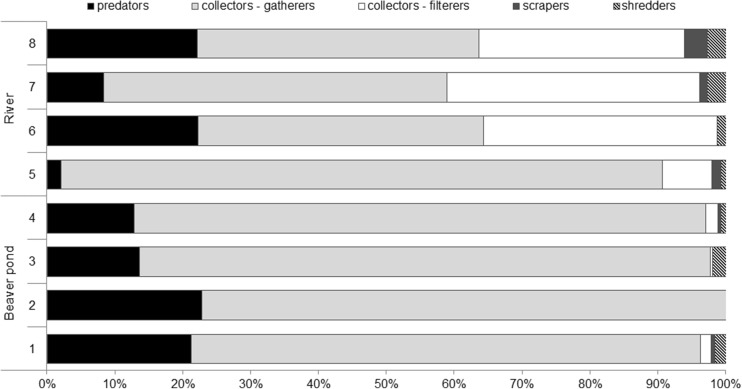
Percentage share of the functional feeding groups of insects in the beaver pond (sites 1–4) and downstream (sites 5–8)

Aquatic insects predominate in the trophic structure of rivers and they were the most abundant group of benthos in the Żylica River and in the beaver pond. In the beaver pond, collectors-gatherers and predators were the most numerous (Fig. 4). A small percentage of shredders and other FFG were also observed. In the beaver pond, only two sites were characterised by the occurrence of five FFG. At site 2, only predators and collectors-gatherers were observed. At all sites, except site 6, in the river all five FFG were present. At the site 5 (below the dam), collectors-gatherers were the most abundant, and at the other river sites, a decrease in their relative abundance was observed along with an increased abundance of collectors-filterers and shredders (Fig. 4).

Analysis of the FFG ratios and metrics indicate that their values were different depending on the site location. All of the ratio values, except for the P/T FFG, were lower in the beaver pond compared to the river.

## Discussion

### Environmental characteristics

The engineering activity of the beavers, disturbs the river continuum after the creation of two new habitat types - beaver ponds and floodplain habitats, as a consequence of the construction of dams (Fustec et al.^2001^; Rolauffs et al., 2001; Harthun 1999; Stringer and Gaywood 2016). This activity converts lotic habitats into lentic habitats. The limitation of the water flow and consequently, the disturbance of the sediment and organic matter transport dynamics, are some of the results of the transformations of a natural ecosystem (Rosell et al. 2005; Arndt and Domdei 2011). The sediments and organic matter content in beaver ponds constitute a substrate for the development of aquatic vegetation (Derwich and Mróz 2008). We did not observe any differences between the organic matter content in the sediments in the beaver pond and in the river, it was high or very high at the sampling sites in both habitats. In this research, aquatic plants were scarce and only a few species occurred at the sampling sites above and below the beaver dam. On the shore, bushy vegetation provided shade over the water in the littoral zone. Beaver dams have a significant impact on the riverine habitats and lead to an increasing heterogeneity of the environments (Gurnell 1998; Collen and Gibson 2000). The creation of the beaver pond on the river Żylica River caused differences in the composition of the benthic taxa groups and the ratio of the FFG as well as an increased invertebrate density compared to the sampling sites located in beaver pond.

Beaver dams modify the geomorphological conditions of streams and riparian areas and affect the biogeochemical processes (Arndt and Domdei 2011; Stout et al. 2017). They also increase the number of animal shelters between the branches of the trees that had been released into the water, thus becoming a link between the water and the environments (Suzuki and McComb 2004). In this research it was impossible to observe the animal shelters between the branches of trees because of the depth and mud sediments next to the dam.

Beavers activity influences the water chemistry (Błędzki et al. 2011; Puttock et al. 2017) and over the years this effect is more visible in the differences between the water parameters in the beaver ponds and in the river water (Collen and Gibson 2000; Law et al. 2017). The construction of the beaver dam did not improve the quality of the water in the Żylica River. The analysis of the water chemistry showed that only the average nitrate content was higher in the river below the dam than in the beaver pond. Only a slight increase in pH was observed. It is likely that the age of the pond and the number of dams play a significant role in obtaining such results. The beaver pond in this study is young and in the Żylica River there is only a single dam. The studies of Devito and Dillon (1993) also identified runoff as an important determinant of nutrient retention in beaver ponds, whereas Ecke et al. (2017), found that beaver ponds constitute a source of phosphorus in the water, but its retention increases with the age of the beaver systems.

### Benthos composition and spatial distribution

As ecosystem engineers, beavers change the structure of the benthos assemblages in the rivers (Duffy and Labar 1994; Margolis et al. 2001; Pliūraitė and Kesminas 2012). The slower water velocity, increased the sedimentation and therefore the presence of a homogeneous substrate may explain the reduced diversity of benthos in the pond (43 taxa) compared to the riverine sampling sites (a total of 55 taxa). The invertebrate assemblages that were found in the pond reflect the changes created by the formation of a new lentic habitat and our results are consistent with the study of Stringer and Gaywood (2016). Six taxa were found only in the beaver pond, whereas 14 taxa were found only in the river. Anderson and Rosemond (2007) as well as Pliūraitė and Kesminas (2012) presented similar results. Baetidae, which are known to be insects less sensitive to the water pollution, were the most numerous in the beaver pond. The composition of the pond benthos was typical for that of eutrophic standing water, marshes or rich aquatic vegetation (Menetrey et al. 2008).

The presence or absence of some groups of invertebrates in the Żylica River is an indication of the specific abiotic and biotic features of an ecosystem that is connected with the beavers activity such as changes in the water velocity, and substratum (Collen and Gibson 2000; Law et al. 2017). Competition, predation, the availability of food, the type of substrate and the oxygen concentration determine the occurrence and abundance of bottom organisms (Pamplin, Almeida, & Rocha, 2006). The composition of the benthos assemblages at the sampling sites located along the river course and in the beaver pond differed. In the downstream part of the river, the diversity of Trichoptera and Coleoptera was greater, while Diptera were more abundant in the beaver pond. In the downstream part of the river, a progressive increase in the proportion of crustaceans was observed. Molluscs and leeches were also more frequent in the river compared to the pond fauna. The observed differences are not only related to the taxonomic composition of the benthos, but also to the structure of its assemblages. They are the consequences of the interactions between both the environmental characteristics and the creation of the beaver pond (Collen and Gibson 2000). Harding et al. Stark (1999) and Dietrich and Anderson (2000) identified a group of factors that have a significant impact on the structure of a benthos group including the substratum type, the surface velocity of the water and depth as factors that determine the diversity of a benthos. Anderson and Rosemond study (2007) showed that beaver ponds had a lower macroinvertebrate taxonomic richness and diversity compared to the downstream parts of a river. Rolauffs et al. (2001), recorded higher benthic densities in a river below a beaver dam and McDowell and Naiman (1986) observed seasonal differences in the density of invertebrates in a beaver pond and a river. While they found that the density of benthos was two to five times higher in a pond than in a stream in spring and summer, they did not find any differences in the density of invertebrates between the two systems in the autumn.

The numerous occurrence of Chironomidae larvae in the pond and below the beaver dam is a consequence of their widespread distribution in many types of water habitats, their ability to adapt to the various physicochemical properties of the water in which they can occupy numerous niches (Bazzanti and Bambacigno 1987; Armitage et al. 1995). They are ecologically important due to their role in nutrient circulation and in food webs (Townsend 2013).

### Functional feeding groups

Analyses of the FFG ratios and metrics indicated that their values were different depending on the site location, meaning that all of the ratio values, except for the P/T FFG, were lower in the beaver ponds compared to the river. According to Merritt et al. (1996), a P/T FFG value of 0.15 indicates a normal top-down predator control. In the beaver pond, this ratio ranged from 0.15 to 0.26, whereas in the river it ranged from 0.02 to 0.28. The value of the ratio CF/GF > 0.50 means that an ecosystem is enriched in suspended particulate organic matter in and such values were only obtained at sites 6, 7 and 8. The values of the SCR + F/ SHR + GC ratio indicate habitat stability when they reach a value of >0.60 (Merritt et al. 1996), which we found only on sites 6, 7 and 8 (in the river). The distribution of the functional feeding group can vary across habitats (Oliveira and Nessimian 2010). The dams that are constructed by beavers can convert a habitat from a lotic to a lentic one, which causes shredders and scrapers to be less abundant while collectors and predators become more abundant (Stringer and Gaywood 2016), which coincides with our findings of the dominance of collectors-gatherers and predators in the beaver pond. In the river sampling sites five FFG were present. Just below the dam, collectors-gatherers were the most abundant and, decreasing percentages were observed along with an increased abundance of collectors-filterers and shredders at the other river sites. Filtering collectors remove the FPOM from the water, while gathering collectors acquire FPOM from the interstices in the bottom sediments (Cummins 1988) and therefore, both are common in the river ecosystems. While shredders chew litter and plant tissue CPOM, scrapers harvest the attached algae from different surfaces (Cummins et al.^2005^). In the study of Arndt and Domdei (2011), they were most abundant in the beaver ponds.

Indices based on Ephemeroptera, Trichoptera and Plecoptera are considered to be useful tools when comparing benthos (Beauger et al. 2006; Uwadiae 2010), because they are good indicators of the dynamics of an ecosystem due to their high sensitivity to environmental disturbances (Pliūraitė and Kesminas 2012), and their values are also used as a measure of stream health. Although the % EPT was visibly greater in the beaver pond, this was the consequence of the large number of Betidae larvae. Some studies have suggested that the removal of the Beatidae family enhances the sensitivity of the EPT index to multiple stressors in streams and rivers (Masese and Raburu 2017), but in fact, EPT is still widely used in studies on river invertebrates (Mahan et al. 2015). A similar result for the number of EPT taxa on comparable habitats was demonstrated by Arndt and Domdei (2011) despite the large number of dragonflies**.** A greater variety of EPT insects is influenced by the local habitat parameters such as the rate of water flow, the substrate type (Anderson and Rosemond 2007) and the presence of plant communities (Wright et al. 2003).

The construction of the dams and the increased water flow below these constructions, creates specific habitats for the species that prefer lotic waters (Clifford et al. 1993; Smith and Mather 2013). The results of our studies showed that the taxonomic changes in the benthic communities were caused by the beaver activity as a consequence of direct factors such as water damming, rather than indirect factors mentioned by Margolis et al. (2001), due to the relatively small changes in the water chemistry and the scarce occurrence of plants. This may also be a consequence of natural variability, the available food and water velocity. Beaver ponds can exist for a long period of time even after the beavers have moved into new lodges. Because these abandoned ponds can still provide a suitable habitat for the occurrence of many invertebrate groups (Rosell et al. 2005), they enrich the local biodiversity.
